# Cellular Effects of Bacterial *N*-3-Oxo-Dodecanoyl-_L_-Homoserine Lactone on the Sponge *Suberites domuncula* (Olivi, 1792): Insights into an Intimate Inter-Kingdom Dialogue

**DOI:** 10.1371/journal.pone.0097662

**Published:** 2014-05-23

**Authors:** Johan Gardères, Joël Henry, Benoit Bernay, Andrès Ritter, Céline Zatylny-Gaudin, Matthias Wiens, Werner E. G. Müller, Gaël Le Pennec

**Affiliations:** 1 Université de Bretagne-Sud, Laboratoire de Biotechnologie et de Chimie Marines, EA 3884, Institut Universitaire Européen de la Mer, Lorient, France; 2 Laboratoire des Mollusques Marins et des Ecosystèmes associés, CNRS INEE FRE 3484, Université de Caen Basse-Normandie, Caen, France; 3 Post Genomic Platform PROTEOGEN, SF ICORE 4206, Université de Caen Basse-Normandie, Caen, France; 4 Pontificia Universidad Católica de Chile - Departamento de Ecología Facultad de Ciencias Biológicas - Santiago - Chile; 5 European Research Council Advanced Investigator Grant Research Group at Institute for Physiological Chemistry, University Medical Center of the Johannes Gutenberg University, Mainz, Germany; Veterinary Pathology, Switzerland

## Abstract

Sponges and bacteria have lived together in complex consortia for 700 million years. As filter feeders, sponges prey on bacteria. Nevertheless, some bacteria are associated with sponges in symbiotic relationships. To enable this association, sponges and bacteria are likely to have developed molecular communication systems. These may include molecules such as *N*-acyl-_L_-homoserine lactones, produced by Gram-negative bacteria also within sponges. In this study, we examined the role of *N*-3-oxododecanoyl-_L_-homoserine lactone (3-oxo-C_12_-HSL) on the expression of immune and apoptotic genes of the host sponge *Suberites domuncula*. This molecule seemed to inhibit the sponge innate immune system through a decrease of the expression of genes coding for proteins sensing the bacterial membrane: a Toll-Like Receptor and a Toll-like Receptor Associated Factor 6 and for an anti-bacterial perforin-like molecule. The expression of the pro-apoptotic caspase-like 3/7 gene decreased as well, whereas the level of mRNA of anti-apoptotic genes Bcl-2 Homolog Proteins did not change. Then, we demonstrated the differential expression of proteins in presence of this 3-oxo-C_12_-HSL using 3D sponge cell cultures. Proteins involved in the first steps of the endocytosis process were highlighted using the 2D electrophoresis protein separation and the MALDI-TOF/TOF protein characterization: α and β subunits of the lysosomal ATPase, a cognin, cofilins-related proteins and cytoskeleton proteins actin, α tubulin and α actinin. The genetic expression of some of these proteins was subsequently followed. We propose that the 3-oxo-C_12_-HSL may participate in the tolerance of the sponge apoptotic and immune systems towards the presence of bacteria. Besides, the sponge may sense the 3-oxo-C_12_-HSL as a molecular evidence of the bacterial presence and/or density in order to regulate the populations of symbiotic bacteria in the sponge. This study is the first report of a bacterial secreted molecule acting on sponge cells and regulating the symbiotic relationship.

## Introduction

Sponges and bacteria have lived in an intimate association for the last 700 million years [1], [2]. This association may be defined as a symbiosis *sensu* De Bary [3]. About 25 bacterial phyla have been identified as being associated with sponges within the *Porifera* phylum [2]. Among them, the *Poribacteria* contains bacteria specifically associated with sponges [2]. Living together, sponges and bacteria have likely developed means to communicate and to regulate the association. For a number of years, research efforts have focused on understanding the relationships between sponge and bacteria. Recent studies demonstrate that some sponge-associated bacterial species possess specific genes coding for proteins with domains found in eukaryotic proteins such as ankyrin, adhesin, fibronectin type III, tetracopeptide repeats, which may be involved in protein-protein interactions in eukaryote organisms [4], [5]. These specific proteins may represent a means for some bacteria to be recognized by sponge cells.

The sponges possess innate immune and apoptotic systems very similar to those of higher vertebrates [6], [7], [8], [9], [10]. The sponge immune system is able to detect bacterial membrane molecules from pathogenic strains and to trigger a differential response [7], [8]. Some proteins involved in the immune pathway have been identified in *Suberites domuncula* and *Amphimedon queenslandica* such as LPS-binding protein [7], TLR (Toll-Like receptor) [9], MyD88 [7], IRAK (Interleukin Receptor Associated Kinase) [9], TRAF 6 (Toll-like Receptor Associated Factor) [10], NF_K_B (Nuclear Factor _K_B) [10] and MPEG (MacroPhage Expressed Gene) [7]. The sponge apoptotic system also involves proteins such as BHP-1 and -2 (BCl-2 homolog proteins) and caspase-like 3/7, which are homologous to proteins from higher vertebrates [9]. The transcription of the caspase-like 3/7 gene increases in the presence of bacterial lipopeptides [9]. Nevertheless, some bacteria are still tolerated by the sponges and are located in contact with or within the cells; they should have found a mean to by-pass or escape these immune and apoptotic reactions. Some bacterial secreted molecules may govern this intimate association.

Bacteria produce a set of different molecules known under the generic name of autoinducers to communicate together. Among these molecules, *N*-acyl-_L_-homoserine lactones (AHLs) are produced by Gram-negative bacteria and regulate cell density-related physiologic events such as biofilm formation, production of virulence factors, or bacterial mobility [11]. Moreover, this family of molecules is able to influence eukaryotic cells. For example, the *N*-3-oxododecanoyl-_L_-homoserine lactone (3-oxo-C_12_-HSL) leads to the disturbance of cell junctions between enterocytes [12] and to the modulation of the immune response [13], [14], [15] in human pathology contexts. Nevertheless, some data demonstrated that AHLs are also involved in bacterial symbiosis processes. Indeed, AHLs produced by *Vibrio fischeri* are accumulated in the light organ of the squid *Euprymna scolopes* and promote the bioluminescence of this bacterium to protect the host [16]. Moreover, AHLs trigger the Ti plasmid transfer process from the bacterium *Agrobacterium tumefaciens* into tobacco root cells to promote the association [17]. But until now, no data showed the direct involvement of AHLs in a direct cross-talk between bacteria and animal cells in a symbiotic context.

The *in vitro* production of AHLs by sponge-associated bacteria has already been documented [18], [19], [20], [21]. Furthermore, these molecules were also detected in crude sponge dicholoromethane extracts, showing that some AHLs (in particular the 3-oxo-C_12_-HSL) are indeed produced within the sponge *S. domuncula* [18]. In order to examine whether 3-oxo-C_12_-HSL could play a role within the sponge as a potential inter-kingdom paracrine factor, we determined the 3-oxo-C_12_-HSL effects on *S. domuncula* cells, a molecule produced *in vitro* and *in vivo* by *S. domuncula*-associated bacteria [18]. This molecule triggered a specific response on the expression of sponge immune and apoptotic genes. Moreover, a proteomic approach demonstrated that some proteins are differently expressed in a 3D cell culture model, the primmorph, in presence of 3-oxo-C_12_-HSL. The characterization of these proteins indicated that sponge cells undergo an endocytosis process.

## Results

### 2.1. Cytotoxicity of 3-oxo-C_12_-HSL on Sponge Cells

The cytotoxicity of 3-oxo-C_12_-HSL was evaluated on sponge cells according to the MTT assay in the presence of 10^−4^ M, 10^−5^ M and 10^−6^ M of the molecule. The non-treated cell cultures were used as a reference. The highest quantity of DMSO (1%) used to dissolve the molecule was also assayed to evaluate the cytotoxic potential of this solvent on sponge cells. No significant difference in cell viability was observed between non-treated and treated cultures (Figure S1). Furthermore, the amount of DMSO used in this experiment did not lead to any difference throughout the length of the experiment, *i.e.* after up to 48 h. The presence of 3-oxo-C_12_-HSL, regardless of the tested concentration, did not induce any difference in the rate of formazan production compared to reference and DMSO-treated cultures.

**Figure 1:**
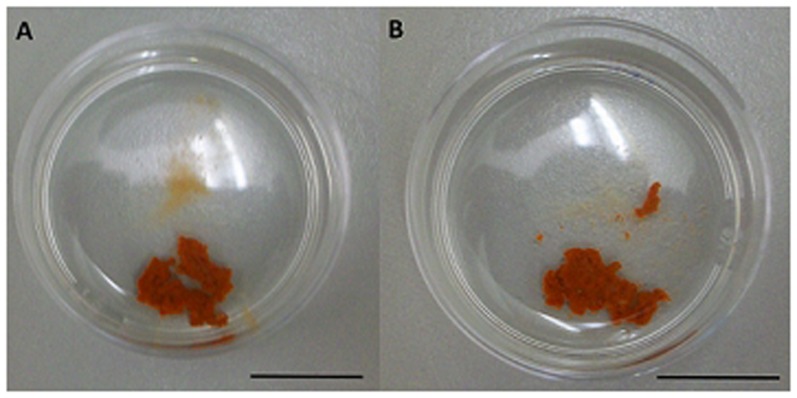
Primmorph cultures stimulated for 48 h with 10^−6^ M of *N*-3-oxododecanoyl-_L_-homoserine (A) or not stimulated (B).

We also examined the morphology of primmorphs in the presence of 3-oxo-C_12_-HSL. Macroscopically, the morphology of primmorphs was not modified after 24 h and 48 h of incubation in the presence of 3-oxo-C_12_-HSL regardless of the concentrations tested (Figure 1). We therefore chose to test the effect of the 3-oxo-C_12_-HSL at the lowest concentration, 10^−6^ M, corresponding to the lowest amount of homoserine lactone found in sponge extracts [18].

### 2.2 Immune and Apoptotic Responses to 3-oxo-C_12_-HSL

**Figure 2:**
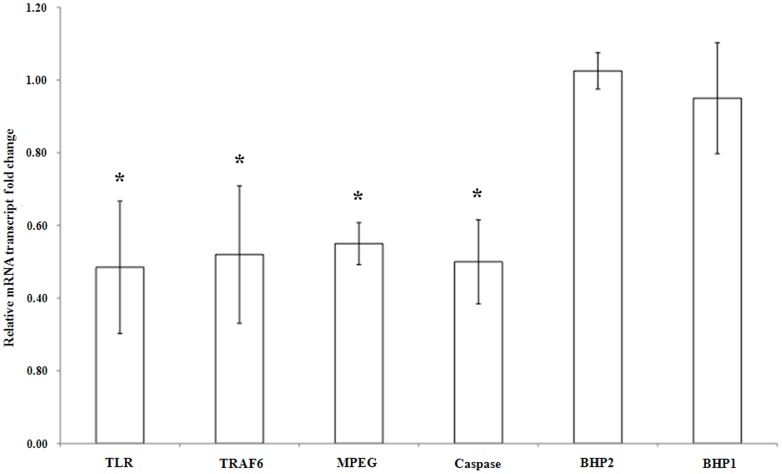
Relative amounts of transcripts for Toll-like receptor (TLR), Toll-like Receptor associated factor 6-like protein (TRAF 6), Macrophage-Expressed Gene protein (MPEG), caspase-like 3/7, BCl-2 homolog protein-1 (BHP-1) and BCl-2 homolog protein-2 (BHP-2) in *N*-3-oxododecanoyl-_L_-homoserine lactone (3-oxo-C_12_-HSL)-stimulated *Suberites domuncula* compared to control *S. domuncula*. The mRNA levels in 8 h-stimulated *S. domuncula* were evaluated by quantitative reverse transcription-PCR and compared to those in control sponges. Reactions were performed in triplicate with three different specimens. Relative mRNA level values resulted from calculating values: values above and below 1 show a higher and a lower mRNA level in the presence of 10^−6^ M 3-oxo-C_12_-HSL, respectively. Significant expression changes (p<0.05) in the presence of *N*-3-oxododecanoyl-_L_-homoserine are pointed out by an asterisk.

The variations in gene expression were studied using quantitative RT-PCR in the presence of 3-oxo-C_12_-HSL for the immune markers: Toll-like receptor, TRAF 6-like and MPEG-like protein, and for the apoptotic markers: caspase 3/7–like, BHP 1 and BHP 2 and statistically examined according to the U-Mann Whitney test (p<0.05). The mRNA levels of the anti-apoptotic genes BHP-1 and BHP-2 did not statistically change in the presence of 10^−6 ^M 3-oxo-C_12_-HSL compared to the control samples, since the average ratios of gene expression are 1 between the treated and the control samples. In contrast, the expression of TLR, TRAF 6-like, MPEG and caspase 3/7-like genes changed statistically in the presence of this AHL. The mRNA levels of these genes decreased on average by a factor of 2 in the presence of 10^−6^ M 3-oxo-C_12_-HSL (Figure 2).

### 2.3 General Protein Response of Sponge Primmorphs to 10^−6 ^M 3-oxo-C_12_-HSL

#### 2.3.1 Total protein fraction

**Figure 3:**
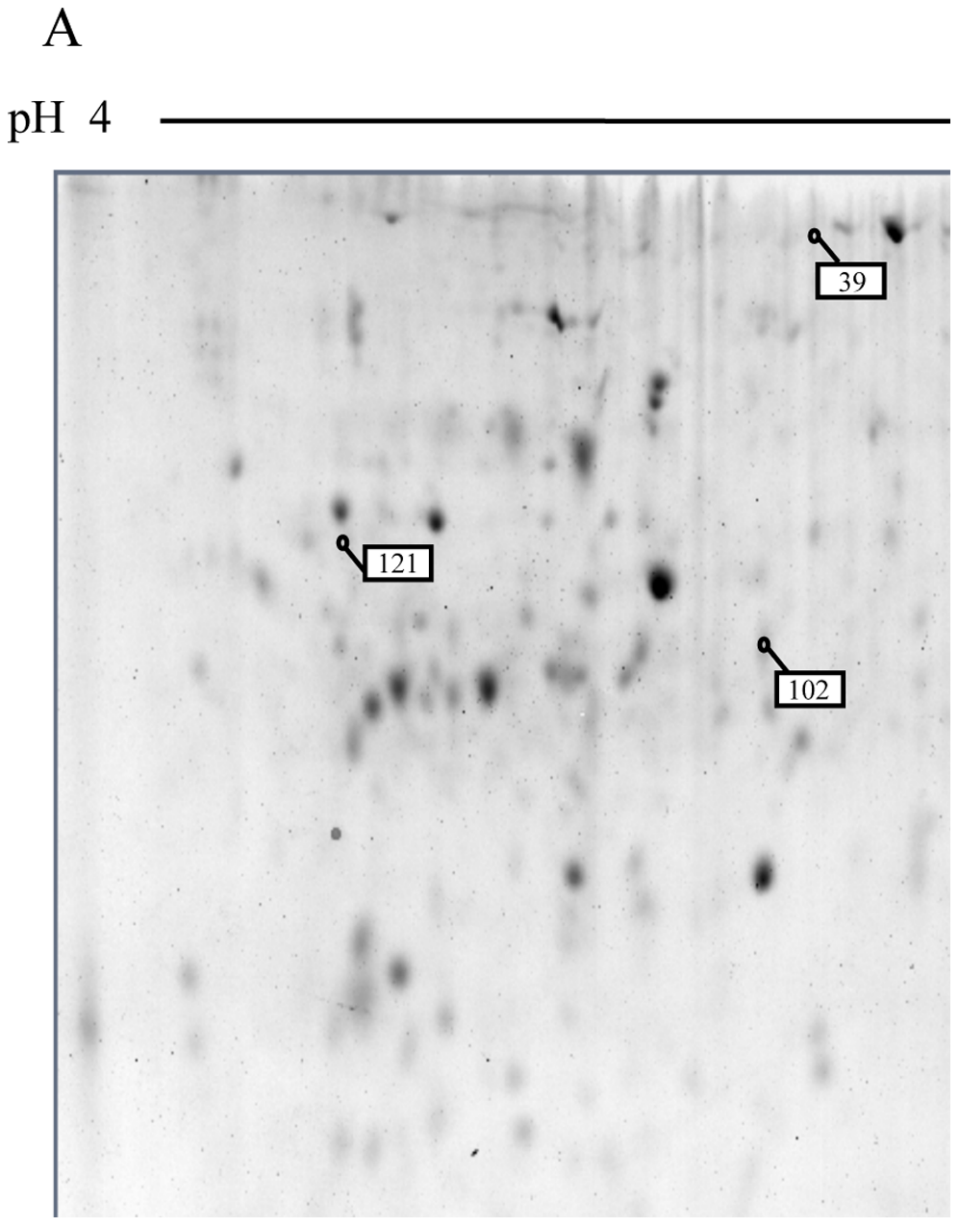
2D electrophoretic profiling of *Suberites domuncula* primmorph extracts in response to 10^−6^ M of *N*-3-oxododecanoyl-_L_-homoserine lactone (3-oxo-C_12_-HSL). Images correspond to representative gels of a total 3-oxo-C_12_-HSL-stimulated protein primmorph extract separated with a linear gradient pH 4–7 (A) or pH 5–8 (B), and of a membrane 3-oxo-C_12_-HSL-stimulated protein primmorph extract separated with a linear gradient pH 5–8 (C). Spots with amounts significantly (p<0.05) changed are highlighted on the gels with white and black boxes for protein, the amount of which is significantly higher or lower in presence of 3-oxo-C_12_-HSL, respectively. Enlargements of these spots are shown in D (left part: 3-oxo-C_12_-HSL -treated sample; right part: untreated sample).

The analysis of 24 h-treated primmorph proteins showed that 12 proteins were over-expressed in the presence of 10^−6 ^M 3-oxo-C_12_-HSL (Figures 3 A, B and C). After characterization and data bank analyses, 7 proteins matched with EST sequences: 5 with *S. domuncula* EST sequences and 2 with *A. queenslandica* EST sequences (Table 1). The alignment of these sequences with the GenBank data base led to the identification of 5 proteins according to their function. Three proteins corresponded to the α and β subunits of a lysosomal ATPase (spots 104, 154 and 157). α actinin (spot 99) and the α subunit of tubulin (spot 169) were also over-expressed in the presence of 10^−6 ^M 3-oxo-C_12_-HSL. Two EST sequences, not identified, were translated and submitted to the Prosite software (spot 17 and 121). No positive match was obtained. However, the protein sequence of spot 121 presented a conserved glutaredoxin domain in N-terminal position (Prosite score = 10.439).

**Table 1:** MS identification of the proteins from total primmorph extract and from the membrane fractions, which displayed differential abundances in *N*-3-oxodecanoyl-_L_-homoserine lactone (3-oxo-C_12_-HSL)-stimulated *Suberites domuncula* primmorphs compared to control primmorphs (p<0.05).

Protein spots		Protein analysis					Protein identification	
protein number regulation^a^		sequencebank^b^	GenBank accession number	Mascot score	recognized peptide numbers	sequence coverage (%)	homology (species)	E-Value
24 h-stimulated primmorphs (total soluble protein fraction)								
17	+	*S. domuncula*	gi|300467112	59	1	11		
39	+							
70	+							
99	+	*S. domuncula*	gi|300479356	93	2	10	α-actinin 1 (*Homo sapiens*)	5. 10^−68^
102	+							
104	+	*S. domuncula*	gi|300478742	167	4	13	α subunit lysosomal ATPase (*Homo sapiens*)	5. 10^−75^
114	+							
121	+	*S. domuncula*	gi|300479590	172	3	16		
143	+							
154	+	*A. queenslandica*	Aqu1.228276	83	3	1	β subunit lysosomal ATPase (*Homo sapiens*)	8. 10^−67^
157	+	*A. queenslandica*	Aqu1.228276	84	4	1	β subunit lysosomal ATPase (*Homo sapiens*)	8. 10^−67^
169	+	*S. domuncula*	gi|300467815	125	3	17	tubulin α-1 chain (*Parentrotus lividus*)	1. 10^−124^
24 h-stimulated primmorphs (membrane soluble protein fraction)								
394	+	*S. domuncula*	gi|300469085	143	4	26	actin (*Aedes aegypti*)	2. 10^−132^
503	+	*S. domuncula*	gi|300465889	74	2	12	cognin (*Gallus gallus*)	5. 10^−58^
1201	+	*S. domuncula*	gi|300465842	78	3	11	cofilin/Actin depolymerizing factor (*Grosmannia clavigera*)	1. 10^−15^
1402	+							
3608	−							
4204	+							
4502	−	*S. domuncula*	gi|300467049	128	4	23	α-tubulin (*Suberites ficus)*	6. 10^−137^
4504	+	*S. domuncula*	gi|300468062	67	1	4	cofilin/Actin depolymerizing factor (*Grosmannia clavigera*)	1. 10^−15^
5202	−							

a The amount of proteins in fractions is higher (+) or lower (−) in presence of 3-oxo-C_12_-HSL.

b Protein MS spectra were compared to theoretical spectra of proteins generated from *S. domuncula* and *Amphimedon queenslandica* EST data bases.

#### 2.3.2 Membrane protein fraction

After statistical analysis of 24 h-treated primmorph proteins, 9 spots were over-represented in the membrane protein fractions either in the presence or in the absence of the 3-oxo-C_12_-HSL (Figures 3 C and D): 6 spots were over-represented in the membranes of 3-oxo-C_12_-HSL-stimulated primmorphs whereas the intensity of 3 spots was higher in the control condition. Five of these characterized proteins matched with *S. domuncula* EST sequences (Table 2). In presence of 10^−6 ^M 3-oxo-C_12_-HSL, an actin, a cognin and 2 cofilins/actin-depolymerizing factors (ADF) were found in higher amounts in primmorph membranes. α tubulin associated to cell membranes was less abundant in the treated samples compared to the control samples. No domain was identified within the four unknown over-represented proteins using the Prosite software.

**Table 2:** Primers used to quantify gene expression in *Suberites domuncula* using real-time PCR.

Genes	forward primers	reverse primers
Toll-like receptor	5′-CGCTTTTACTTCAGCTGCCC-3′	5′-CCTGCGGGACCAAAGAACAAG-3′
Caspase 3/7 -like	5′-AACTGTTCCGAAATCAGGCG-3′	5′-GTTGATGTCTGGCGACGTATG-3′
TRAF6-like	5′-GGAATCGCACAAACTGGCTT-3′	5′-CAGCCAAGATCAGCAAGTGG-3′
α ATPase	5′-GGCCTTTTATAACCAGGCCC-3′	5′-TCAGACTGCGAGGTGTGCTC-3′
actinin α_1_	5′-GAGCTCAGCATGCAAGGTCA-3′	5′-GGCTTCAAGTTGGTCCTCCA-3′
tubulin α	5′-GCATGGAATCCAACCAGACG-3′	5′-CCGCCAAGGGTTTTGTCA-3′
spot 121	5′-AGCACCCTGGCAAGTCAGAG-3′	5′-TGCCAGCCTCCTTCTCCATA-3′
HPRT	5′-TACTGGAGCCACGATGACCAAG-3′	5′-TGGTCTGTATCCCACACTGAGG-3′
GAPDH	5′-TCCAAACCAGCCAAGTACGATG-3′	5′-AGTGAGTGTCTCCCCTGAAGTC-3′

### 2.4 Sponge mRNA Levels of Genes Encoding the Differentially Primmorphs Expressed Proteins

**Figure 4:**
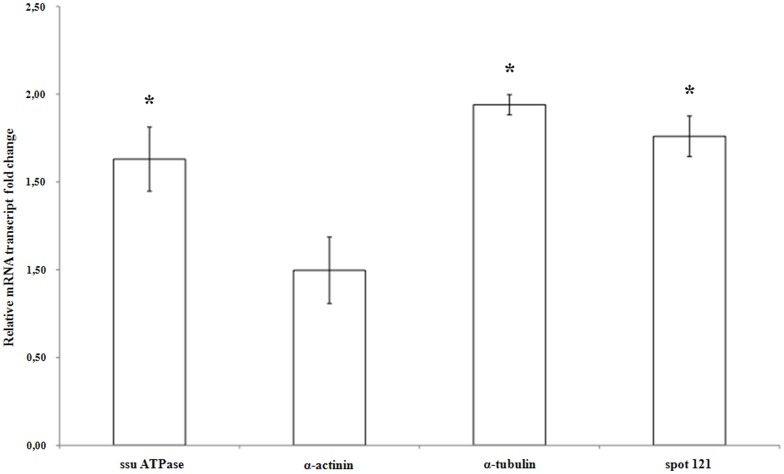
Relative amounts of the α subunit of ATPase (ssu a ATPase), α actinin, α tubulin and protein spot 121 transcripts in *N*-3-oxododecanoyl-_L_-homoserine lactone (3-oxo-C_12_-HSL)-stimulated *Suberites domuncula* compared to the control. The mRNA levels in stimulated S. *domuncula* were evaluated by quantitative reverse transcription-PCR and compared to non-stimulated sponges. Reactions were performed in triplicate with three different specimens. Relative mRNA level values resulted from calculating values: values above and below 1 show a higher and a lower mRNA level in the presence of 10^−6^ M 3-oxo-C_12_-HSL, respectively. Significant expression changes (p<0.05) in the presence of *N*-3-oxododecanoyl-_L_-homoserine are pointed out by an asterisk.

The mRNA levels of genes encoding the differentially expressed *S. domuncula* proteins, α actinin, α ATPase, α tubulin and the protein from spot 121, were compared in the sponge organism stimulated or not by 10^−6 ^M 3-oxo-C_12_-HSL for 8 h. The values shown in Figure 4 represent the average ratio between the expressions of each gene in presence of the AHL *versus* in the control condition. Significant differences were examined according to the U-Mann Whitney test (p<0.05). α actinin mRNA level did not vary statistically in the presence or in the absence of this AHL. The expression of genes encoding the α ATPase, the α tubulin and the spot 121 protein increased 1.63, 1.94 and 1.76 fold, respectively, in the presence of 10^−6 ^M 3-oxo-C_12_-HSL (Figure 4). The statistical analysis showed that their expression differed significantly in the presence of 10^−6^ M 3-oxo-C_12_-HSL.

## Materials and Methods

### 3.1 Live Material, Primmorph Culture, and Stimulation

*Suberites domuncula* is the sponge used in this study. No specific authorization is requested to collect sponges in open areas for scientific purposes. This study did not involve endangered or protected species.

Live specimens were collected along the Brittany coasts (Bay of Brest, Brittany, France) by scuba diving (at a water depth of 5 m) and kept alive in an artificial seawater aquarium at 11°C. The physical and chemical parameters were controlled twice a week prior to feeding [22]. For each experiment, sponge cells were dissociated individually from 3 different specimens for cell culture purposes [18], [22]. Briefly, a piece (2 cm^2^) of each sponge specimen was cut individually into small cubes (1 mm^3^) in calcium- and magnesium-free seawater (CMFSW) (460 mM NaCl, 7 mM Na_2_SO_4_, 10 mM KCl, 10 mM Hepes, 73 µg mL^−1^ of penicillin G potassium, 34.5 µg mL^−1^ of streptomycin sulfate, pH 7.4) containing EDTA (250 mM) under sterile conditions. The resulting fragments were transferred into a 50-mL conical tube and incubated at room temperature (RT°C, namely 20°C ±1) on a rotary shaker for 30 min. The supernatant was filtered through a nylon cloth with pores of 20 µm diameter. Fresh CMFSW-EDTA solution was added on the remaining pieces of sponge, and the suspension was incubated once again on the rotary shaker for 30 min. The resulting filtered cell suspensions were centrifuged at 800 g at RT°C for 5 min. The supernatants were discarded, and the cells were suspended twice in CMFSW to remove the EDTA. Finally, the cells from the two incubations were pooled and centrifuged at 800 g at RT°C for 5 min. The cell pellet was then suspended in 0.2 µm-filtered natural seawater collected at seashore at Ploemeur, Brittany, France. The seawater was supplemented by sea sand (Sigma-Aldrich, France), 10 µM ferrous citrate (Sigma-Aldrich, France), 1 mM of pyruvate (Sigma-Aldrich, France) and 73 µg mL^−1^ of Penicillin G potassium and 34.5 µg mL^−1^ of Streptomycin sulfate to prevent bacterial growth. The cell suspension was separated in two fractions of equal volumes, and both were incubated into Petri dishes, at 15°C under permanent gentle shaking. During the first two days of incubation, the cells clumped and aggregated, forming axenic primmorphs. At this stage, two identical cultures from the same sponge were available. After 3 days of culture, the culture medium was completely renewed with fresh culture medium without antibiotic. Old culture medium was spread on Marine growth agar (Difco, France) to check for bacterial growth. In order to study early *de novo* proteins, three-day-old primmorphs were stimulated for 24 h at 15°C under smooth shaking conditions, either with 10^−6^ M 3-oxo-C_12_-HSL (Sigma-Aldrich, France) dissolved in DMSO (Sigma-Aldrich, France) 0.1% (v: v) or with DMSO 0.1% (v: v) as a control. After the stimulation, the primmorphs were directly used for further experiments or stored dried at −80°C.

### 3.2 Cytotoxicity Assays

The cytotoxic assays were performed according to Zhang *et al*. (2004) [23] in triplicate with cells from two different specimens. After tissue dissociation, the sponge cells were cultured in 96 well plates into supplemented seawater. Briefly, 200 microliters of the cell suspension (10^7^ cells mL^−1^) were laid in each well and incubated for 16 h at 15°C. The cells were then incubated for 24 h and 48 h in presence of 3-oxo-C_12_-HSL (10^−4^ M, 10^−5^ and 10^−6^ M) or DMSO 0.1% (v: v). Cell cultures without AHL or DMSO supplementation were used as a reference.

Cell viability was determined with a solution of tetrazolium compound (3-(4,5-dimethylthiazol-2-yl)-2,5-diphenyl tetrazolium) (MTT) (Sigma-Aldrich, France). Twenty microliters of a MTT solution (5 mg mL^−1^) were added into each culture well and incubated for 2 h at 15°C. After centrifugation at 800 g for 10 min at 4°C, the supernatant was removed and replaced by 200 µL DMSO. The plate was shaken in the darkness and then sonicated to promote the cell lysis. The production of formazan, which is related to cell viability [24], was determined by measuring its absorbance at 540 nm with a spectrophotometric 96-well plate reader (Tecan, France). A U-Mann Whitney test was performed as a statistical test with the following hypothesis: the results are different between the controls and the samples (p<0.05) using the Statistica software (StatSoft, France).

### 3.3 Preparation of Total and Membrane Proteins

Protein extractions were performed on primmorphs. Primmorphs were collected by centrifugation at 800 g for 5 min at 4°C and washed with CMFSW. Primmorphs were then crushed using a Polytron homogenizer in 2 mL of lysis solution (1% Triton 100X, 20 mM Tris-HCl, 3 mM MgCl_2_, 1 mM EDTA, 0.15 mM NaCl and 1 mM PMSF, pH 7.4) and further incubated on ice for 2 h under gentle agitation. The mixture was then centrifuged at 400 g for 10 min at 4°C. The supernatant was collected and precipitated overnight at −20°C by 4 volumes of precipitating solution (0.1 M ammonium acetate, acetone 80% (v: v)) in order to pellet the total protein fraction. For membrane preparation, an additional step was added. The centrifuged lysate was laid onto a saccharose gradient (10, 15, 35 and 40% (w: v)) and centrifuged at 200,000 g for 15 h at 4°C. The 15% fraction was collected and precipitated as described above. The precipitate was collected after centrifugation at 10,000 g for 20 min at 4°C. The protein pellet was subsequently washed twice in ice-cold acetone. The pellet was quickly dried at RT°C and suspended in a protein solubilization buffer (7M Urea, 2M Thiourea, CHAPS 4% (w: v), 60 mM DTT, 10 mM Tris-HCl, protease inhibitor mix (GE Healthcare, France), pH 7.4). A final cleaning step was added using the 2-D Clean-Up Kit (Bio-Rad Laboratories, France). The total protein concentration was assayed by using the Quick Start Bradford (Bio-Rad Laboratories, France). The extracts were stored at −80°C.

#### 3.4 2-D protein separation

For the three replicates of each condition, either for the total proteins or for the membrane proteins, 600 µg of proteins were solubilized into 300 µL of the solubilization buffer supplemented by 1X Biolytes, pH 3-10 (Bio-Rad Laboratories, France) and 0.005% (w: v) bromophenol blue. The protein solution was loaded onto a 17 cm ReadyStrip, pH range 4–7 or 5–8 (Bio-Rad Laboratories, France) according to the manufacturer’s instructions. After an overnight active rehydration step at 50 V in the Protean IEF Cell (Bio-Rad Laboratories) thermo-regulated at 20°C, the isoelectrofocalisation, the SDS-PAGE, and the gel staining were performed as described in the MIAPE GE (Table S1).

### 3.5 Image Analysis

Gels were scanned using the Molecular Imager G800 Calibrated Densitometer (Bio-Rad Laboratories, France) at 400 dpi by Quantity One software (Bio-Rad Laboratories, France). The gels were then analyzed with the Melanie software (GE Healthcare, France). Manual editing and normalization were applied after automated spot detection and matching. Spot quantification was based on spot density as a percentage of the total spot density of the gel to normalize for possible staining differences between gels. Gel annotations and matching fidelity were checked manually to eliminate matching errors caused by the software. All spots selected for MS analyses presented statistically significant variations (p<0.05) using the U-Mann Whitney test (Statistica software, (StatSoft, France).

### 3.6 In Gel Digestion and Protein Analysis

The spots of interest were manually excised from the gel. The gel pieces were washed individually for 30 min in 200 µL of solution A (ammonium bicarbonate and acetonitrile mix: 2.5 mL of 1 M ammonium bicarbonate added to 47.5 mL water and 50 mL acetonitrile). Washings with ammonium bicarbonate 25 mM and solution A were repeated until the gel was discolored. The subsequent washings were performed with 200 µL of ultrapure HPLC grade water, then with 200 µL of 100% HPLC grade acetonitrile. Each washing lasted approximately 15 min at RT°C under shaking. The supernatants were discarded and the gels were dried using a vacuum pump for 5 min at RT°C. Twenty-five microliters of a diluted trypsin solution (0.006 µg µL^−1^ in 25 mM ammonium bicarbonate) were added to each gel piece and rehydration was done on ice for 15 min. Thirty microliters of 25 mM ammonium bicarbonate were added to complete the rehydration, and the samples were incubated overnight at 37°C under continuous shaking. The supernatant was discarded, and the peptides were extracted for 15 min successively with 30 µL of a solution of 50% (v: v) of acetonitrile in water, 30 µL of a 5% formic acid (v: v) solution in water, and finally with 30 µL 100% acetonitrile. The solutions were gathered together prior to drying using a vacuum pump or a dry bath at 55°C until complete evaporation. The sample was stored at −20°C until analysis.

### 3.7 Mass Spectrometry and Bank Search

MS experiments were carried out on an AB Sciex 5800 proteomics analyzer equipped with TOF TOF ion optics and an OptiBeam on-axis laser irradiation with 1000 Hz repetition rate (AB Sciex, USA). The system was calibrated immediately before analysis with a mixture of des-Arg-Bradykinin, Angiotenin I, Glu1-Fibrinopeptide B, ACTH (18–39), ACTH (7–38) and a mass precision less than 5 ppm. 0.8 µL of the peptide solution was mixed with 1.6 µL of α-cyano-4-hydroxycinnamic acid (CHCA) matrix dissolved in a solution of 50% acetonitrile supplemented with 0.1% TFA (v: v). The mixture was spotted on a stainless steel Opti-TOF 384 targets (Applied Biosystems, USA); the droplet was evaporated before introducing the target into the mass spectrometer. All acquisitions were taken in automatic mode. A laser intensity of 3,000 A was typically used for ionizing. MS spectra were acquired in the positive reflector mode by summarizing 1000 single spectra (5×200) in the m/z 700–4,000 range. MS/MS spectra were acquired in the positive MS/MS reflector mode by summarizing a maximum of 2,500 single spectra (10×250) with a laser intensity of 3,900 A. For the tandem MS experiments, the acceleration voltage applied was 1 kV, and air was used as the collision gas. The fragmentation pattern was used to determine the sequence of the peptide. Database searching was performed using the Mascot 2.3.02 program (Matrix Science, USA). Two Expressed Sequence Tags (EST) databases corresponding to updated compilation downloaded from the NCBI database were used with the demosponges *S. domuncula* and *A. queenslandica* as selected species (including 13,684 and 30,060 entries respectively). The variable modifications allowed were as follows: N-terminal acetylation, methionine oxidation, and dioxidation. “Trypsin” was selected as enzyme, and three miscleavages were also allowed. Each matching cDNA sequence was submitted to the NCBI protein data base (blastx) to find a potential homology with known proteins of other species. Homologous proteins possessing an E-value lower than 10^−10^ were considered to possess the same identity as the studied protein. The sequences of unknown proteins were then analyzed by Scanprosit (expasy.org/tools/scanprosite) to predict putative functions for the proteins under study.

### 3.8 Quantitative Reverse Transcription-PCR (qRT-PCR) Analysis

Three sponge specimens were separated in two pieces, incubated in 100 mL of 0.2 µm-filtered seawater and stimulated for 8 h either by 10^−6^ M 3-oxo-C_12_-HSL or by 0.1% DMSO (v: v) as control sample, respectively. Eight hours were chosen according to Smith et al. (2001) [25]. Total RNAs were isolated from fresh sponge tissues in the extraction solution RNA now (Epicentre, USA), and DNAse (Epicentre, USA) treated according to the manufacturer’s instructions. Five hundred nanograms of total RNAs were used to synthesize first-strand cDNAs using oligodT primers with MultiScribe Reverse Transcriptase (Applied Biosystem, USA) according to the manufacturer’s instructions. A negative control was included using total RNA without the MultiScribe Reverse Transcriptase. The mRNAs of interest were quantified by real-time PCR amplification of their cDNAs. The relative mRNA transcript change of each gene was studied for immune markers: Toll-like receptor, TRAF 6-like and MPEG-like protein, and for apoptotic markers: caspase 3/7–like, BHP 1 and BHP 2. Primers (Table 2) were designed with Primer Express 3 software (Applied Biosystems, USA) in order to have a melting temperature (Tm) between 58 and 60°C. Each primer pair was validated by verifying that the PCR efficiency E was above 0.95, and that a single PCR product with the expected Tm was obtained. PCR reactions were performed in triplicate with the 7300 Real Time PCR System apparatus (Applied Biosystems, USA). The 20 µL reactions contained 10 µL SYBR Green PCR Master Mix (including AmpliTaq Gold DNA Polymerase) (Applied Biosystems, USA), 2 µL of each primer (3 µM) and 8 µL of cDNAs. For each experiment, three serial cDNA dilutions were used: 0.25, 0.025 and 0.0025 ng µL^−1^. The PCR conditions were 95°C for 10 min for polymerase activation, and 40 cycles at 95 and 60°C for 60 and 30 s, respectively. The relative quantification of the mRNAs of interest was obtained by the comparative Pfaffl method using HPRT or GAPDH as endogenous control (Table 2) [26]:
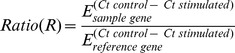
E: efficiency of the primer couple, Ct: cycle threshold. Relative mRNA level values resulted from calculated values: values above and below 1 showed a higher and a lower mRNA level in the presence of 10^−6^ M 3-oxo-C_12_-HSL, respectively (*e.g.*, a value of 1.5 indicated a 1.5-fold increased mRNA level in stimulated condition; and a value of 0.66 revealed that the mRNA level was divided by 1.5 in stimulated condition).

A U-Mann Whitney test was performed as a statistical test with the following hypothesis: the relative transcript level ratios are different between the controls and the samples (p<0.05), using the Statistica software (StatSoft, France).

## Discussion

Sponges and bacteria have been associated within consortia for approximately 700 million years [1], [2]. As foreign organisms, on the one hand, the bacteria need to find a means to protect themselves from the host chemical defense or predation and, on the other hand, the host needs to sense the population of bacteria to discriminate between the pathogenic and the non-pathogenic ones and to regulate the density of resident bacteria. Both organisms are able to express molecules to communicate between themselves such as quorum-sensing molecules for the bacteria [11] and hormone-like factors for sponges [27]. Some of these molecules may be able to act as cross-kingdom dialog factors. Among these potential molecules, AHLs are produced *in vivo* by bacteria within the sponge *S. domuncula* [18]. These secreted molecules, produced by Gram-negative bacteria, were recognized as acting on eukaryotic cells of higher vertebrates [12], [13], [14], [15]. Nevertheless, these studies have always been performed in pathological contexts, and never in a symbiotic one *sensu* De Bary [3]. The purpose of this study was to investigate the potential of 3-oxo-C_12_-HSL, an AHL which was found in crude sponge extracts [18] and in culture supernatants of sponge-associated bacteria [18], [19], [20], [21], as a symbiotic paracrine factor in the sponge bacterium consortium.

As a xenobiotic molecule, 3-oxo-C_12_-HSL may trigger an answer of the immune system and/or the apoptotic pathway, both present in this Porifera phylum [6], [7], [8], [9], [10]. In order to avoid a cross-contamination by bacteria established in the sponge, axenic 3-D cultures (primmorphs) were used. Primmorphs were first incubated in presence of 10^−6^ M 3-oxo-C_12_-HSL for 48 h. This primary contact did not influence the shape or the color of the primmorphs maintained *in vitro*, indicating that 3-oxo-C_12_-HSL was devoid of a deleterious effect at the macroscopic level. The cell cytotoxicity has been also evaluated: 3-oxo-C_12_-HSL at 10^−4^, 10^−5^ and 10^−6^ M, as well as the solvent DMSO (1%), did not have any significant cytotoxic effect on sponge cells. These results were in agreement with those observed on human polynuclear neutrophile cells at 10^−5^ and 10^−4^ M [4].

The different experiments performed on eukaryotic cells with 3-oxo-C_12_-HSL showed that this molecule can have a negative impact on the immune and apoptosis systems of the host cells. For example, the bacterium *Pseudomonas aeruginosa* uses this molecule to promote its installation and proliferation into the lungs or the intestine. Indeed, 3-oxo-C_12_-HSL modifies the integrity of the barrier formed by human intestinal epithelial cells [12] and is able to inhibit the vasoconstriction of blood vessels in the heart and lungs [4]. It blocks the proliferation of leucocytes [13] and the differentiation of human T lymphocytes [14]. This molecule also induces the apoptosis in human T lymphocytes by activating the mitochondrial apoptotic pathway [15]. 3-oxo-C_12_-HSL is also able to influence the secretion of cytokine by immune cells [30], [31], [32], [33]. Sponges possess an innate immune system and an apoptotic pathway close to those of higher eukaryotes [6], [7], [8], [9], [10]. They are also able to sense and to respond differently to Gram-positive or Gram-negative bacteria [6], [7], [8]. In order to examine the 3-oxo-C12-HSL effects on sponge immunity and apoptosis, the expression level of genes was compared between primmorphs stimulated or not by 10^−6^ M 3-oxo-C_12_-HSL for 8 h. The expression levels of the TRAF 6-like protein, the TLR and the perforin-like MPEG were monitored for the immune system and the one of the caspase 3/7-like, the BHP 1 and the BHP 2 for the apoptotic system. After 8 h of stimulation, the presence of 3-oxo-C_12_-HSL led to reduced mRNA levels of the genes encoding the TLR, the TRAF 6-like protein, the MPEG and the caspase 3/7-like. The expression of the anti-apoptotic BHP 1 and BHP 2 genes did not seem to be influenced by this bacterial molecule. The *S. domuncula* TLR shares homologies with vertebrate TLR 1 and 6 [9]. It is able to sense lipopeptides, and its activation by these molecules triggers the increase of the caspase 3/7-like gene transcription in the sponge *S. domuncula* [9]. The TRAF 6 protein is an ubiquitin ligase involved in several vertebrate cell pathways [34], [35], [36], [37]. It triggers the transcription of a lot of vertebrate pro-inflammatory genes *via* the activation of the NFκB transcription factors and of the p38 and Jun N-terminal Kinases (JNK) [37]. In the sponge *S. domuncula*, Böhm et al. (2001) demonstrated that these same pathways are activated in the presence of *Escherichia coli* lipopolysaccharides (LPS) [38]. The MPEG, which possesses an antibacterial activity, is also over-expressed in the presence of *E. coli* LPS [7]. 3-oxo-C_12_-HSL may inhibit, at the tested concentration, the expression of the genes coding for factors involved in pathways of bacterial membrane molecule detection and in antibacterial factor production. 3-oxo-C_12_-HSL also reduced the expression of the caspase 3/7-like gene, whereas it did not affect the expression of anti-apoptotic BHP1 and BHP2 genes. The expression of this caspase 3/7-like gene is stimulated by a lipopeptide found in Gram-negative and Gram-positive bacterial membranes [9]. The present result suggests that 3-oxo-C_12_-HSL at 10^−6^ M inhibits the apoptotic response of sponge cells. So, by expressing 3-oxo-C_12_-HSL, inhabiting sponge-associated bacteria may reduce the immune and apoptotic responses of sponge cells. This may suggest that this AHL, probably in coordination with others, could participate in the takeover of the eukaryotic cell by the pathogenic bacteria to their own benefits. The sponge may also “interpret” this molecule to limit its own immune and apoptotic response when its choanocytes feed on bacteria.

The study of the general response to 3-oxo-C_12_-HSL was performed in order to elucidate the main response triggered by this molecule. This differential analysis of 2D-electrophoretic protein pattern has been carried out for the first time on sponges. *S. domuncula* primmorphs were used in order to prevent the unwanted bacterial response to the bacterial molecule. Consistently, the results did not show any differentially expressed bacterial protein. The expression of genes, whose proteins were differentially expressed in primmorph total protein extracts, was investigated in the whole consortium bacteria-sponge to prove any difference in the way of regulation.

In the presence of 10^−6^ M 3-oxo-C_12_-HSL, some proteins were differentially expressed in the total sponge protein extracts. The protein spot 121, over-expressed in presence of 3-oxo-C_12_-HSL, has not been identified but possesses a glutaredoxin domain. Its gene is also over-expressed after 8 h in the presence of this molecule. This kind of protein, often involved in detoxification processes, is also over-expressed in the plant *Medicago truncatula* in the presence of two AHLs [39]. Moreover, glutaredoxin-1 is involved in the activation of the TRAF-6 protein in Hela cells, which is bound to a S-glutathione group in its inactive state. This mechanism promotes the activation of the IL-1 R/TLR 4 pathway [40]. The sponge may use this protein to try to activate its immune system and/or to eliminate 3-oxo-C_12_-HSL. Some subunits of the lysosomal ATPase, the α and β subunits, were over-expressed, forming the catalytic site of ATPase [41]. Two spots with different isoelectric points corresponded to the same β subunit. This difference may be explained by posttranslational modifications encountered in phosphorylated proteins [42]. 3-oxo-C_12_-HSL at 10^−6^ M stimulates the expression of the lysosomal α ATPase gene after 8 h of incubation with sponges. Nevertheless, over-expressed cytoskeleton associated-proteins have also been identified: an α actinin and an α tubulin. 3-oxo-C_12_-HSL stimulates the transcription of the α tubulin gene after 8 h, whereas this molecule did not affect the transcription of the α actinin gene. The increase of a posttranslational form of this protein in the presence of 3-oxo-C_12_-HSL may explain the higher amount of this protein in 3-oxo-C_12_-HSL-stimulated primmorph extracts. The incubation time may also be inappropriate to observe the effect of the molecule on the gene expression. The genes transcription observed in whole individual varied in a same manner as the expression of proteins observed in primmorph in the presence of 10^−6^ M 3-oxo-C_12_-HSL. A correlation between the level of genes expression and the corresponding protein is difficult to establish since regulation processes of the transcription and the regulation of proteins are independent in eukaryotes. Furthermore, the gene expression level between the sponge and the primmorph may also be somehow different. In the presence of 10^−6^ M 3-oxo-C_12_-HSL, some proteins were also differentially recruited to the sponge cell membranes. Actin and 2 cofilins/ADF proteins were recruited to the membranes, whereas the α tubulin seems to be more represented in the cytoplasm of 3-oxo-C_12_-HSL stimulated-sponge cells.

The proteins identified in this study correspond to proteins involved in the first steps of endocytosis [43], [44]. Indeed, the α actinin is a protein involved in the link between the cell membrane and the intracellular actin. In higher vertebrates, this protein is recruited during the activation of integrins [45], participating for example to the bacteria phagocytosis [43], [44]. It links the β subunit of the integrin to actin and participates in the engulfment of bacteria by the cell. Moreover, β integrin homolog sequences have been discovered in the genome of the sponge *A. queenslandica* [10]. The lysosomal ATPase is recruited very early in this process directly in the phagosomes of mouse macrophages. It is transported by tubular lysosomes, formed by α tubulin, and contributes to the acidification of early phagosomes [45]. Cofilin/ADF proteins are adjoined to the membrane during the phagosome formation. They regulate the polymerization of actin filaments around the *Dictostelium* phagosome [47]. Nevertheless, these proteins are also involved in the internalization of the parasite *Listeria monocytogenes* [48], [49]. A cognin-like protein is also recruited to the membrane in the presence of 3-oxo-C_12_-HSL. This membrane protein intervenes in cell aggregation thanks to its disulfide isomerase activity, which creates temporary covalent links with membrane proteins of the other cells [50], [51]. Some data demonstrate that some sponge-associated bacteria possess proteins with eukaryotic protein-protein interaction domains [5]. The cognin-like protein may intervene in the capture of these bacteria.

3-oxo-C_12_-HSL seems to stimulate the *de novo* protein expression as well as the recruitment of proteins involved in the internalization of bacteria and further in the first step of the endocytosis/phagocytosis process. Indeed, the over-expression of lysosomal V-ATPase, early recruited to the phagosomes via tubular lysosomes to establish the hostile acidic environment [46], suggests that 3-oxo-C_12_-HSL promotes the phagocytosis process in the sponge cells. In the same way, the phagocytosis activity is stimulated in response to 3-oxo-C_12_-HSL in immune cells of higher vertebrates [28], [29], [52]. This increase of activity due to this molecule may correspond to an ancestral mechanism conserved in the evolution. The 3-oxo-C_12_-HSL effects on the plant *M. truncatula* also lead to a modification of the expression of cytoskeletal protein such as α tubulin and a cofilin/ADF protein [39].

To conclude, 3-oxo-C_12_-HSL may participate in the control of the immune and apoptotic *S. domuncula* systems. This takeover does not presume of the destiny of bacteria in the eukaryotic cells: taking advantage like in pathological contexts or long-lasting installation like in symbiosis. But the sponge may perceive this signal as a molecular evidence of the bacterial presence/density and may then trigger the first steps of bacterial endocytosis and detoxification mechanism in order to control its bacterioflora. Indeed, this mechanism may lead the sponge to react to the multiplication of the inhabiting bacteria in order to regulate the population. This secreted 3-oxo-C_12_-HSL is the first molecule demonstrating a paracrine inter-kingdom action in the sponge and in a non-pathological context. These results open up a new perspective in the understanding of the mechanism of regulation of sponge/bacterium relationships.

## Supporting Information

Figure S1 **Effect of** ***N*** **-3-oxododecanoyl-_L_-homoserine lactone (3-oxo-C_12_-HSL) on the viability of** ***Suberites domuncula*** **cells.** Histogram of the optical density of the cell lysate at 540 nm corresponding to the amount of formazan formed by the alive *S. domuncula* cells. Sponge cells were cultured and then stimulated with 10^−6^ (soft grey), 10^−5^ (medium grey) and 10^−4^ M (dark grey) 3-oxo-C_12_-HSL, with 1% DMSO (v: v) (white) and with only culture medium (black) for 24 h and 48 h.
(TIF)

Table S1 Complementary information regarding the proteomic experiments.
(XLS)

## References

[1] Müller WE, Wang X, Schröder HC (2009) Paleoclimate and evolution: emergence of sponges during neoproterozoic. Prog Mol Subcell Biol 47: 55–77. 19198773 10.1007/978-3-540-88552-8_3

[2] Schmitt S, Tsai P, Bell J, Fromont J, Ilan M, et al (2011) Assessing the complex sponge microbiota: core, variable and species-specific bacterial communities in marine sponges. ISME J 6: 564–576. 21993395 10.1038/ismej.2011.116 PMC3280146

[3] De Bary HA (1879) Die Erscheinung der Symbiose. Privately printed in Strasburg.

[4] Siegl A, Kamke J, Hochmuth T, Piel J, Richter M, et al (2011) Single-cell genomics reveals the lifestyle of Poribacteria, a candidate phylum symbiotically associated with marine sponges. ISME J 5: 61–70. 20613790 10.1038/ismej.2010.95 PMC3105677

[5] Thomas T, Rusch D, Demaere MZ, Yung PY, Lewis M, et al (2010) Functional genomic signatures of sponge bacteria reveal unique and shared features of symbiosis. ISME J 4: 1557–1567. 20520651 10.1038/ismej.2010.74

[6] Wiens M, Perovic-Ottstadt S, Müller IM, Müller WE (2004) Allograft rejection in the mixed cell reaction system of the demosponge *Suberites domuncula* is controlled by differential expression of apoptotic genes. Immunogenetics 56: 597–610. 15517243 10.1007/s00251-004-0718-6

[7] Wiens M, Korzhev M, Krasko A, Thakur NL, Perovic-Ottstadt S, et al (2005) Innate immune defense of the sponge *Suberites domuncula* against bacteria involves a MyD88-dependent signaling pathway. Induction of a perforin-like molecule. J Biol Chem 280: 27949–27959. 15923643 10.1074/jbc.M504049200

[8] Thakur N, Perovic-Ottstadt S, Batel R, Korzhev M, Diehl-Seifert B, et al (2005) Innate immune defense of the sponge *Suberites domuncula* against gram-positive bacteria: induction of lysozyme and AdaPTin. Mar Biol 146: 271–282.

[9] Wiens M, Korzhev M, Perovic-Ottstadt S, Luthringer B, Brandt D, et al (2007) Toll-like receptors are part of the innate immune defense system of sponges (demospongiae: Porifera). Mol Biol Evol 24: 792–804. 17190971 10.1093/molbev/msl208

[10] Srivastava M, Simakov O, Chapman J, Fahey B, Gauthier ME, et al (2010) The *Amphimedon queenslandica* genome and the evolution of animal complexity. Nature 466: 720–726. 20686567 10.1038/nature09201 PMC3130542

[11] Reading NC, Sperandio V (2006) Quorum sensing: the many languages of bacteria. FEMS Microbiol Lett 254: 1–11. 16451172 10.1111/j.1574-6968.2005.00001.x

[12] Vikstrom E, Tafazoli F, Magnusson KE (2006) *Pseudomonas aeruginosa* quorum sensing molecule *N*-(3 oxododecanoyl)-_L_-homoserine lactone disrupts epithelial barrier integrity of Caco-2 cells. FEBS Lett 580: 6921–6928. 17157842 10.1016/j.febslet.2006.11.057

[13] Bryan A, Watters C, Koenig L, Youn E, Olmos A, et al (2010) Human transcriptome analysis reveals a potential role for active transport in the metabolism of *Pseudomonas aeruginosa* autoinducers. Microbes Infect 12: 1042–1050. 20659582 10.1016/j.micinf.2010.07.006 PMC2963707

[14] Ritchie AJ, Jansson A, Stallberg J, Nilsson P, Lysaght P, et al (2005) The *Pseudomonas aeruginosa* quorum-sensing molecule *N*-3-(oxododecanoyl)-_L_-homoserine lactone inhibits T-cell differentiation and cytokine production by a mechanism involving an early step in T-cell activation. Infect Immun 73: 1648–1655. 15731065 10.1128/IAI.73.3.1648-1655.2005 PMC1064928

[15] Jacobi CA, Schiffner F, Henkel M, Waibel M, Stork B, et al (2009) Effects of bacterial *N*-acyl homoserine lactones on human Jurkat T lymphocytes-OdDHL induce apoptosis via the mitochondrial pathway. Int J Med Microbiol 299: 509–519. 19464950 10.1016/j.ijmm.2009.03.005

[16] McFall-Ngai MJ, Montgomery MK (1990) The anatomy and morphology of the adult bacterial light organ of *Euprymna scolopes* Berry (Cephalopoda: Sepiolidae). Biol Bull 179: 332–339. 29314961 10.2307/1542325

[17] Zhang L, Murphy PJ, Kerr A, Tate ME (1993) *Agrobacterium* conjugation and gene regulation by *N*-acyl-_L_-homoserine lactones. Nature 362: 446–448. 8464475 10.1038/362446a0

[18] Gardères J, Taupin L, Bin Saïdin J, Dufour A, Le Pennec G (2012) *N*-acyl homoserine lactone production by bacteria within the sponge *Suberites domuncula* (Olivi, 1792) (Porifera, Demospongiae). Mar Biol 159: 1685–1692.

[19] Zan J, Cicirelli EM, Mohamed NM, Sibhatu H, Kroll S, et al (2012) A complex LuxR-LuxI type quorum sensing network in a roseobacterial marine sponge symbiont activates flagellar motility and inhibits biofilm formation. Mol Microbiol 85: 916–933. 22742196 10.1111/j.1365-2958.2012.08149.x PMC3429658

[20] Taylor MW, Schupp PJ, Baillie HJ, Charlton TS, de Nys R, et al (2004) Evidence for acyl homoserine lactone signal production in bacteria associated with marine sponges. Appl Environ Microbiol 70: 4387–4389. 15240326 10.1128/AEM.70.7.4387-4389.2004 PMC444791

[21] Mohamed NM, Cicirelle EM, Kan J, Chen F, Fuqua C, et al (2008) Diversity and quorum-sensing signal production of Proteobacteria associated with marine sponges. Environ Microbiol 10: 75–86. 18211268 10.1111/j.1462-2920.2007.01431.x

[22] Le Pennec G, Perovic S, Ammar MS, Grebenjuk VA, Steffen R, et al (2003) Cultivation of primmorphs from the marine sponge *Suberites domuncula*: morphogenetic potential of silicon and iron. J Biotechnol 100: 93–108. 12423904 10.1016/s0168-1656(02)00259-6

[23] Zhang X, Le Pennec G, Steffen R, Müller WE, Zhang W (2004) Application of a MTT assay for screening nutritional factors in growth media of primary sponge cell culture. Biotechnol Prog 20, 151–155. 10.1021/bp0341601 14763838

[24] Mosmann T (1983) Rapid colorimetric assay for cellular growth and survival: application to proliferation and cytotoxicity assays. J Immunol Methods 65: 55–63. 6606682 10.1016/0022-1759(83)90303-4

[25] Smith RS, Fedyk ER, Springer TA, Mukaida N, Iglewski BH, et al (2001) IL-8 production in human lung fibroblasts and epithelial cells activated by the Pseudomonas autoinducer N-3-oxododecanoyl homoserine lactone is transcriptionally regulated by NF-kappa B and activator protein-2. J Immunol 167: 366–74. 11418672 10.4049/jimmunol.167.1.366

[26] Pfaffl MW (2001) A new mathematical model for relative quantification in real-time RT-PCR. Nucleic Acids Res 29: e45. 11328886 10.1093/nar/29.9.e45 PMC55695

[27] Müller WE, Wiens M, Müller IM, Schroder HC (2004) The chemokine networks in sponges: potential roles in morphogenesis, immunity and stem cell formation. Prog Mol Subcell Biol 34: 103–143. 14979666 10.1007/978-3-642-18670-7_5

[28] Wagner C, Zimmermann S, Brenner-Weiss G, Hug F, Prior B, et al (2007) The quorum-sensing molecule *N*-3-oxododecanoyl homoserine lactone (3OC12-HSL) enhances the host defense by activating human polymorphonuclear neutrophils (PMN). Anal Bioanal Chem 387: 481–487. 16906383 10.1007/s00216-006-0698-5

[29] Lawrence RN, Dunn WR, Bycroft B, Camara M, Chhabra SR, et al (1999) The *Pseudomonas aeruginosa* quorum-sensing signal molecule, *N*-(3-oxododecanoyl)-_L_-homoserine lactone, inhibits porcine arterial smooth muscle contraction. Br J Pharmacol 128: 845–848. 10556916 10.1038/sj.bjp.0702870 PMC1571710

[30] Smith RS, Harris SG, Phipps R, Iglewski B (2002) The *Pseudomonas aeruginosa* quorum-sensing molecule *N*-(3-oxododecanoyl)homoserine lactone contributes to virulence and induces inflammation in vivo. J Bacteriol 184: 1132–1139. 11807074 10.1128/jb.184.4.1132-1139.2002 PMC134808

[31] Smith RS, Fedyk ER, Springer TA, Mukaida N, Iglewski BH, et al (2001) IL-8 production in human lung fibroblasts and epithelial cells activated by the *Pseudomonas* autoinducer *N*-3-oxododecanoyl homoserine lactone is transcriptionally regulated by NF-kappa B and activator protein-2. J Immunol 167: 366–74. 11418672 10.4049/jimmunol.167.1.366

[32] Telford G, Wheeler D, Williams P, Tomkins PT, Appleby P, et al (1998) The *Pseudomonas aeruginosa* quorum sensing signal molecule *N*-(3-oxododecanoyl)-_L_-homoserine lactone has immunomodulatory activity. Infect Immun 66: 36–42. 9423836 10.1128/iai.66.1.36-42.1998 PMC107855

[33] Hooi DS, Bycroft BW, Chhabra SR, Williams P, Pritchard DI (2004) Differential immune modulatory activity of *Pseudomonas aeruginosa* quorum-sensing signal molecules. Infect Immun 72: 6463–6470. 15501777 10.1128/IAI.72.11.6463-6470.2004 PMC522992

[34] Wong BR, Besser D, Kim N, Arron JR, Vologodskaia M, et al (1999) TRANCE, a TNF family member, activates Akt/PKB through a signaling complex involving TRAF6 and c-Src. Mol Cell 4: 1041–1049. 10635328 10.1016/s1097-2765(00)80232-4

[35] Heldin CH, Landstrom M, Moustakas A (2009) Mechanism of TGF-beta signaling to growth arrest, apoptosis, and epithelial-mesenchymal transition. Curr Opin Cell Biol 21: 166–176. 19237272 10.1016/j.ceb.2009.01.021

[36] Landstrom M (2010) The TAK1-TRAF6 signalling pathway. Int J Biochem Cell Biol 42: 585–589. 20060931 10.1016/j.biocel.2009.12.023

[37] Tang L, Zhou XD, Wang Q, Zhang L, Wang Y, et al (2011) Expression of TRAF6 and pro-inflammatory cytokines through activation of TLR2, TLR4, NOD1, and NOD2 in human periodontal ligament fibroblasts. Arch Oral Biol 56: 1064–1072. 21457942 10.1016/j.archoralbio.2011.02.020

[38] Böhm M, Hentschel U, Friedrich AB, Fieseler L, Steffen R, et al (2001) Molecular response of the sponge *Suberites domuncula* to bacterial infection. Mar Biol 139: 1037–1045.

[39] Mathesius U, Mulders S, Gao M, Teplitski M, Caetano-Anolles G, et al (2003) Extensive and specific responses of a eukaryote to bacterial quorum sensing signals. Proc Natl Acad Sci USA 100: 1444–1449. 12511600 10.1073/pnas.262672599 PMC298792

[40] Chantzoura E, Prinarakis E, Panagopoulos D, Mosialos G, Spyrou G (2010) Glutaredoxin-1 regulates TRAF6 activation and the IL-1 receptor/TLR4 signalling. Biochem Biophys Res Commun 403: 335–339. 21078302 10.1016/j.bbrc.2010.11.029

[41] Sun-Wada GH, Wada Y, Futai M (2003) Lysosome and lysosome-related organelles responsible for specialized functions in higher organisms, with special emphasis on vacuolar-type proton ATPase. Cell Struct Funct 28: 455–463. 14745137 10.1247/csf.28.455

[42] Qiang F, Peng-Cheng L, Jin-Xing W, Qi-Sheng S, XiaoFan Z (2009) Proteomic identification of differentially expressed and phosphorylated proteins in epidermis involved in larval-pupal metamorphosis of *Helicoverpa armigera* . BMC Genomics 10: 600. 20003373 10.1186/1471-2164-10-600 PMC2806347

[43] Allen LA, Aderem A (1996) Mechanisms of phagocytosis. Curr Opin Immunol 8: 36–40. 8729444 10.1016/s0952-7915(96)80102-6

[44] May RC, Machesky LM (2001) Phagocytosis and the actin cytoskeleton. J Cell Sci 114: 1061–1077. 11228151 10.1242/jcs.114.6.1061

[45] Otey CA, Pavalko FM, Burridge K (1990) An interaction between alpha actinin and the beta 1 integrin subunit *in vitro* . J Cell Biol 111: 721–729. 2116421 10.1083/jcb.111.2.721 PMC2116186

[46] Sun-Wada GH, Tabata H, Kawamura N, Aoyama M, Wada Y (2009) Direct recruitment of H^+^-ATPase from lysosomes for phagosomal acidification. J Cell Sci 122: 2504–2513. 19549681 10.1242/jcs.050443

[47] Aizawa H, Fukui Y, Yahara I (1997) Live dynamics of *Dictyostelium* cofilin suggests a role in remodeling actin latticework into bundles. J Cell Sci 110: 2333–2344. 9410873 10.1242/jcs.110.19.2333

[48] Brieher WM, Kueh HY, Ballif BA, Mitchison TJ (2006) Rapid actin monomer-insensitive depolymerization of *Listeria* actin comet tails by cofilin, coronin, and Aip1. J Cell Biol 175: 315–324. 17060499 10.1083/jcb.200603149 PMC2064572

[49] Bamburg JR (2011) *Listeria monocytogenes* cell invasion: a new role for cofilin in coordinating actin dynamics and membrane lipids. Mol Microbiol 81: 851–854. 21762221 10.1111/j.1365-2958.2011.07759.x

[50] Pariser HP, Rakeman AS, Hausman RE (1998) Thioreductase activity of retina cognin and its role in cell adhesion. Brain Res Dev Brain Res 111: 1–9. 9804865 10.1016/s0165-3806(98)00113-8

[51] Pariser HP, Zhang J, Hausman RE (2000) The cell adhesion molecule retina cognin is a cell surface protein disulfide isomerase that uses disulfide exchange activity to modulate cell adhesion. Exp Cell Res 258: 42–52. 10912786 10.1006/excr.2000.4931

[52] Vikstrom E, Magnusson KE, Pivoriunas A (2005) The *Pseudomonas aeruginosa* quorum-sensing molecule *N*-(3-oxododecanoyl)-_L_-homoserine lactone stimulates phagocytic activity in human macrophages through the p38 MAPK pathway. Microbes Infect 7: 1512–1518. 16039899 10.1016/j.micinf.2005.05.012

