# Supplementary figures and images for: Cellular Effects of Bacterial N-3-Oxo-Dodecanoyl-L-Homoserine Lactone on the Sponge Suberites domuncula (Olivi, 1792): Insights into an Intimate Inter-Kingdom Dialogue
Source: PLoS One. 2014 May 23;9(5):e97662. doi: 10.1371/journal.pone.0097662 (PMC4032237; doi:10.1371/journal.pone.0097662)

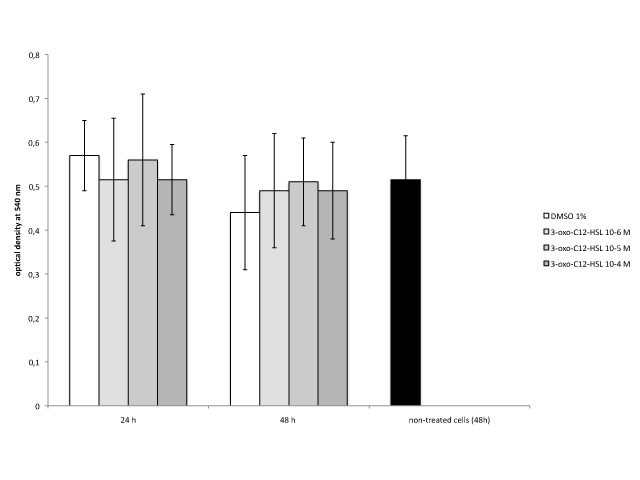

Supplement: Figure S1 — Effect of N -3-oxododecanoyl-L-homoserine lactone (3-oxo-C12-HSL) on the viability of Suberites domuncula cells. Histogram of the optical density of the cell lysate at 540 nm corresponding to the amount of formazan formed by the alive S. domuncula cells. Sponge cells were cultured and then stimulated with 10−6 (soft grey), 10−5 (medium grey) and 10−4 M (dark grey) 3-oxo-C12-HSL, with 1% DMSO (v: v) (white) and with only culture medium (black) for 24 h and 48 h. (TIF) [file pone.0097662.s001.tif]
